# ROLE OF LIFESTYLE IN THE DEVELOPMENT OF CHRONIC OBSTRUCTIVE PULMONARY DISEASE: A REVIEW

**DOI:** 10.4103/0970-2113.59591

**Published:** 2008

**Authors:** Surya Kant, Barkha Gupta

**Affiliations:** 1 Professor Dept. of Pulmonary Medicine, Chhtrapati Shahuji Maharaj Medical University (Erstwhile King George Medical University, Lucknow; 2 Research Scholar Dept. of Pulmonary Medicine, Chhtrapati Shahuji Maharaj Medical University (Erstwhile King George Medical University, Lucknow

**Keywords:** COPD, smoking, indoor pollution, ETS

## INTRODUCTION

Phenomenal increase in population during the last fifty years has led to rapid industrialization and high rate of urbanization. This has created tremendous burden on natural resources. Overcrowding and inadequate housing, unplanned location of the industries in the urban and sub urban areas and traffic congestion, have lead to deterioration of environment with consequent adverse effects on health of mankind. Lack of gainful employment in villages and ecological stress is leading to an ever-increasing movement of the poor families to towns. Mega cities are emerging and urban slums are expanding. The relationship between urbanization and industrialization is complex as it operates in both the directions and is mediated by the socio political factors, environment and disease exposure. One of the major impacts of this transformation is “Westernization” leading to a change in the lifestyle pattern. At the start of the new millennium, the pace and complexity of the life seems to be increasing exponentially. While the penetration and influence of modern communications, technology and economic systems related to what is termed as “globalization” have been a dominant theme since the late twentieth century, there seems to have a confluence of changes in these factors that have led to a major global concern about the rapid globalization of the world economy and its impact on various sub populations. As a result, the urban elites in the developing world are experiencing higher rate of non-communicable diseases, that demand medical therapy of the kind, found in affluent societies i.e. high technology & hospital based medical care, thus escalating the cost of health care.

In 1997, Murray and Lopez has published the results of Global Burden of Disease Study^1^ and found that in the last two or three decades, there has been profound shift in the major cause of mortality worldwide with non communicable chronic diseases such as cancer, Cardio Vascular Disease, stroke, chronic obstructive pulmonary disease (COPD) and diabetes mellitus accounting for more than two-third of the deaths. The leading causes of death and disability and the risk factors that cause them their global ranking, and their distribution by broad region, is shown in Figure I Further, in both developing and developed countries alcohol, tobacoo, and dietary factors were found to be the main causes of disease burden. Table I summarizes the contribution of selected risk factors for the leading diseases causing deaths. The increasing prevalence of COPD, thus is, not an exception to be associated with the development of “Western lifestyle”. Chronic obstructive pulmonary disease, which includes chronic bronchitis and emphysema^2^–^5^, is a progressive disease characterized by airflow limitation/obstruction that is either not reversible at all or only partially reversible. The airflow obstruction in COPD is associated with abnormal inflammatory response of the lungs to chronic inhalational exposure from smokes, dusts and gases. COPD includes chronic obstructive bronchiolitis with fibrosis and obstruction of small airways and emphysema with enlargement of air spaces and destruction of lung parenchyma, loss of lung elasticity and closure of small airways.. Most patients with COPD have all the three pathological mechanisms (chronic obstructive bronchiolitis, emphysema and mucus plugging) as all are induced by smoking but may differ in proportion of emphysema and obstructive bronchiolitis^6^.

**FIG I:**
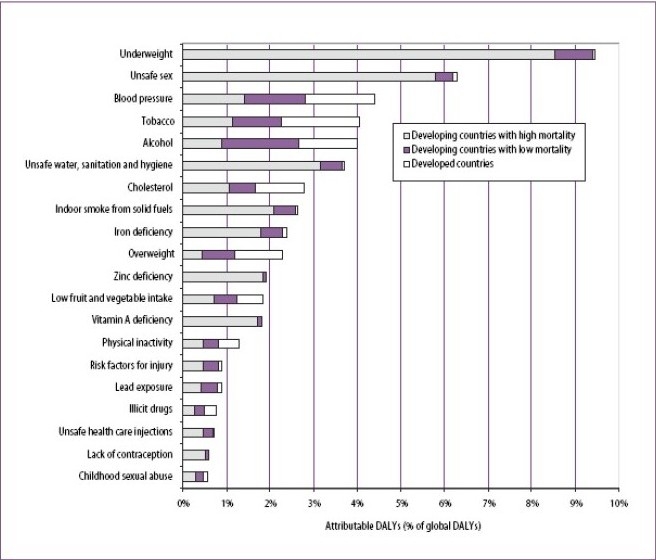
Global Burden of Disease attributable to 20 leading selected risk factors **Source:** World Health Report 2002

**Table 1:** Individual and joint contribution of selected risk factors to leading cause of deaths

	Contributing risk factors (individual PAF for disease burden) (%)							

Diseases	Smoking	Alcohol	High Blood Pressure	High Choles terol	High BMI	Low fruit/vegetable intake	Physical inactivity	Combined PAF%
Lung cancer	85*	-	-	-	-	11	-	86
Cerebrova-								
scular Diseases	22	0	72	27	23	12	9	81-86
Ischemic heart disease	22	0-2	58	63	33	28	22	89-93
Chronic obstructive								
lung disease	85	0	-	-	-	11	-	71
Road accidents	-	38	-	-	-	-	-	-

**Source:** Modified from Ezzati et at Global Burden of disease study Lancet 1997

Ageing of population is not the only cause of the increased prevalence of COPD observed in the industrialized countries but other risk factors must be taken into account. The tobacco smoking epidemic, which hit the developed countries, especially among young people and subsequently among women, and spreading in other countries, is the most important cause. Table II summarizes the risk factors involved in the causation of COPD, either singly or interacting among themselves.

**Table 2:** Trend in % of COPD mortality from smoking worldwide during the period 1985-1995 (age 5-69 years)

Country	COPD 1985 (%)	COPD 1995 (%)
Belgium	75	69
Switzerland	68	64
Finland	68	62
Greece	67	62
Austria	65	65
Netherlands	73	73
Italy	71	70
France	57	63
Portugal	45	55
Spain	59	64
Norway	58	65
Germany	63	69
Russia	72	75
Canada	76	78
United States	78	80
Japan	47	50
Australia	69	66
New Zealand	71	71

**Source:** World Development Indicators, World Bank, 1988

Cigarette smoking is by far the commonest cause of COPD but there are other modifiable risk factors including air pollution (particularly indoor air pollution from burning fuels) and occupational exposures. Table III gives an account for preventable risk factors due to change in the lifestyle associated with the development of COPD.

**Table 3:** Risk factors associated with the development of COPD

Degree of Certainty	Environmental factors
Supposed	Adenovirus infection
	Dietary deficiency of Vitamin C
	Indoor air pollution

Good evidence	Outdoor air pollution
	Low socioeconomic status
	Alcohol intake
	ETS in childhood
	Other occupation exposures

Certain	Tobacoo smoke
	Some occupational exposures

**Source:** ERS Consensus Statement 1995

## A] SMOKING

Smoking is the detrimental risk factor in the development and progression of COPD. Trends in worldwide mortality of COPD from smoking during the period 1985-1995 are shown in the Table IV Emphysema, a major component of COPD, is thought to be due to an excess of proteases, causing destruction of elastin and collagen matrix supporting the lung structure. Tobacco smoking causes an influx of neutrophils into the lungs and a subsequent release of elastase and proteases. Oxidants inhaled from tobacco smoke and released from the activated inflammatory cells play a role in the development of emphysema by impairing endogeneous antiproteases.

**Table 4:** Preventable risk factors associated with the development of COPD

A] Smoking	I. Tobacco smoking	Cigratte
		Bidi
	II. EnvironmentalTobacco smoke ETS	Maternal smoking
		Passive smoking
B] Work exposure or Occupational dust		
C] Air pollution	I. Indoor pollution	
	II. Outdoor pollution	
D] Diet		
E] Alcohol		
F] Socio economic status		

A large number of people, men and women, smoke for pleasure, relaxation, relief of tension or for a sense of security. Among the many social changes recorded in the industrialized world, smoking behavior is explained as social conditioning and social desirability. By smoking, a smoker subconsciously projects the image of prosperity, power or potency.

While smoking rates are declining among men in some countries, it is rising among women worldwide. There were large increases in smoking in developing countries, especially among males, over the last part of the 20th century^7^,^8^. This contrasts with the steady but slow decrease, mostly among men, in many industrialized countries. Smoking rates remain relatively high in most former socialist economies. While prevalence of tobacco use has declined in some high-income countries, it is increasing in some low and middle income countries, especially among young people and women. By the year 2025, the number of women smoker is expected to triple. Women born in United States have the highest level of smoking (32%) compared to South European (20%) and Asian born women (21%) Growing participation of women in active professional life has increased the number of smokers. Industry marketing and advertising strategies are particularly targeted to women and young girls. “Feminine” brands emphasizing low tar, length and slimness have successfully played on traditional fear of weight gain. This has been attributed to the fact that in Scotland rate of COPD have almost doubled amongst women in past 10 years^9^.

The growing trends of tobacco consumption amongst children is due to increasing marketism and consumerism along with Westernisation. Unlike adults, children do not buy cheapest cigarettes, they buy the trendiest, most advertised brands. Tobacoo advertising is intended to increase the consumption as well as brand and has a powerful effect on young people^10^.

### Cigarette:

Cigarette smokers, have higher prevalence of lung function abnormalities and respiratory symptoms, a greater annual rate of decline in FEV_1_ and higher death rate from COPD as compared to non smokers. The decline in FEV_1_ in smokers was proved in a longitudinal study in UK^11^. Pipe and cigar smokers have higher COPD mortality and morbidity rates than non smokers, although it was lower than that of cigarette smokers^12^.

### Bidi:

In India alone, bidi consumption is 900 billions sticks are smoked per year. The amount of tobacco used in bidis is twice to that used in bidi^13^. Bidi smoking is thus more likely to cause clinical and functional impairment of lungs as compared to cigarette smoking^14^.

## II ENVIRONMENTAL TOBACCOO SMOKE (ETS)

Environmental tobacco smoke (ETS) constitutes a common problem in many countries. Passively inhaled tobacco smoke contains several known and probable human carcinogens, as well as irritants and toxic substances^15^.

Passive smoking to cigarette smoke may also contribute to respiratory symptoms and COPD by increasing the lungs total burden of inhaled particulars and gases^16^–^18^. The Canadian Human Time-Activity Pattern Survey has shown that exposure to ETS is common among children, particularly in their homes^19^. Recent studies have shown significant effects from ETS on the occurrence of chronic respiratory symptoms in adults, but only in a limited number of studies has exposure to ETS actually been associated with COPD in adults and with a small impairment of lung function.

### Maternal smoking

Results from animal studies showed that fetal lung development was adversely influenced by maternal smoking This suggested that prenatal exposure to substances inhaled by the smoking mother may be a risk factor for COPD^20^. Maternal smoking is related to lung function deficits in neonates^21^. Postnatal exposure to tobacco smoke conveys increased risks of lower respiratory infections and reduced lung function^22^,^23^. Smoking during pregnancy affects the lung growth and development in uterus and possibly the priming of immune function^24^,^25^. Infants of smoking parents have more respiratory illness than infants of non smoking parents^26^.

## B] WORK EXPOSURE OR OCCUPATIONAL DUST

For many years, researchers have known that chronic exposure to fumes, chemical substances, and dusts in the work place are one of the main risk factors for the development of COPD. The most important are grain, isocyanates, cadmium, coal and other mineral dusts, heavy metals, adhesives and welding fumes. The table IV shows the list of agents associated with COPD. Rapid population growth and urbanization has led to lack of services such as supply of food, water and housing. Lack of employment in villages and the ecological stress has led leading to an ever-increasing movement of poor families to town. Industrialization occurs when a nation's economic system decreases its reliance on hand made goods and increases its reliance upon producing goods by machine. With this, occupational health hazards are also increasing.

**Table 5:** Occupational risk factors for COPD

**Agents with good evidence for cause of COPD**
Cadmium
Silica
**Jobs with increased risk of COPD**
Coal miners
Construction/Cement
Furnace/metal workers/heat exposure
Transport
Grain/farmers
Wood/paper
Cotton
**General population**
Dust exposed
Fume exposed (risk less than that of dust exposure)

**Source:** G.Veigi Epidemilogy of COPD Respiration 2001, 68:4-19

When the exposures are sufficiently intense or prolonged, occupational dust and chemicals (vapors, irritants and fumes) can cause COPD independently of cigarette smoking. It increases the risk of the disease in presence of concurrent cigarette smoking^27^. There is now growing that airway obstruction (COPD) is caused by other exposures rather than tobacco smoke alone, and that occupational exposures, particularly dusts, are important amongst such causes. The widespread habit of tobacco smoking, in industrial population also has delayed the recognition of the other factors contributing the disease.

Farmers, grain workers, construction or cement exposed workers; foundry workers, wood workers and workers exposed to excess heat including furnace workers have been identified as the increased risk groups. Longitudinal studies of the effects of occupational exposures have been performed in coal miners^28^ hard-rock miners^29^ tunnel workers^30^ concrete-manufacturing workers^31^ and in a subject of nonmining industrial workers in Paris^32^. Grain dust exposure also has been established as a risk factor in COPD, both in smokers and nonsmokers^33^.

Development of disease is influenced by the amount of exposure and the toxicity of the dust, and the disease is characterized by long latency periods; therefore, even in countries in which exposures have been recognized and controlled, the disease rates are only gradually declining^34^. Rate trends in developing countries are mostly unknown but the magnitude of the problem is substantial^35^.

## C] AIR POLLUTION

## a) OUTDOOR POLLUTION

Air pollution is another important risk factors for COPD. Chronic exposure to elevated air pollution seems to correlate with chronic bronchitis and lung function impairment. The populations of the rapidly expanding mega cities of Asia, Africa and Latin America are increasingly exposed to levels of ambient air pollution that rival and often exceed those experienced in industrialized countries in the first half of the 20th century^36^.

The factors accounting for the deteriorating urban air quality are growing industrialization and increasing vehicular traffic. Industrial emissions, automobile exhaust and the burning of fossil fuels leads to respiratory damage, heart disease and lung diseases.

Conventional outdoor pollutants includes fossil fuel smoke, sulphur dioxide, nitrogen dioxides and ozone. Air pollution has worsened due to traffic congestion, poor housing, poor sanitation, and drainage and garbage accumulation. A study reported related photochemical oxidants and multiple primary air pollutants such as sulfur dioxide particles and hydrocarbons to chronic respiratory symptoms and pulmonary function abnormalities in both smokers and nonsmokers^37^.

As shown in Table VI the mortality impacts of air pollution using vital statistics for 1991, deaths would increase by 1,385 in Delhi if TSP were to increase by 100 micrograms, whereas the Schwartz and Dockery coefficient for total nontrauma deaths predicts an increase of 3,524 deaths.

**Table 6:** Selected Occupational Agents Associated With COPD

**Gases**	Sulfur dioxide
**Minerals**	Coal
	Man made vitreous fibres
	Oil mist
	Portland cement
	Silica
	Silicates
**Metals**	Cadmium
	Vanadium
	Osmium
	Welding fumes
**Organic dusts**	Cotton
	Endotoxins
	Grain
	Wood
**Smoke**	Internal combustion engine exhaust
	Environmental tobacoo smoke
	Fire smoke

**Source:** John R.Balmes Work Related COPD PCCU Volume 18, Lesson I

**Table 7:** Mortality Impacts of Air Pollution

Mortality End point	Percent Increase in Mortality per 100 *μ*gm/m3 increase in TSP	
	Delhi^1^	Philadelphia (Schwartz & Dockery)
By Selected Cause:		
Total Deaths	2.3 *	6.7 *
CVD	4.3 *	9.2 *
Respiratory	3.1 *	Pneumonia: 10.2
		COPD : 17.8 *

* Indicates significance at 95% confidence

1 Poisson Model : with trigonometric terms, weather, year and trends

**Source:** Schwartz and Dockery 1991

## b) INDOOR POLLUTION

Household energy and indoor air pollution pose a substantial vulnerability to the health of rural women and children. The highest concentration of indoor air pollutants emerges from burning of biofuels such as wood, agriculture crops and dung cake, which are extensively used by rural households in India. It has been estimated that approximately half the global population and up to 90 percent of rural households in developing countries still rely on biomass fuels^38^ and about 75 per cent of Indian households use biofuels for cooking purposes. Typically cooked indoors in open fires or poorly functioning stoves, which leads to levels of air pollution that are among the highest ever measured. Therefore indoor air pollution with biofuels is an issue that requires to be addressed through gender, energy, environment and health policy. Some of highest concentrations of pollutants come about due to the use of biofuels for cooking in rural indoor environment^39^. In developed countries transformation has without exception been come with by a shift from biofuel to petroleum products (kerosene, LPG) and electricity. In developing countries even where cleaner more sophisticated fuels are available, households often continue to use biomass^40^. Although the portion of global energy derived from biofuel has fallen substantially which is evidence that biofuel use is increasing among the poor. Poverty is one of the main hurdles to the adoption of cleaner fuels and slow pace of development in many countries implies that biofuels will continue to be used by the poor for many years.

In addition to passive smoking, which is well known for its harmful action on respiratory health, a number of compounds and mixtures have been identified as relevant air pollutants indoors^41^. These may be derived from heating, combustion, photochemical reactions, furniture, building materials, biological organisms and fibers The smoke from combustion of solid such as wood, dried dung and crop residue used for cooking and heating is the significant cause of indoor air pollution. Over-crowding and inadequate housing conditions contribute to indoor air pollution and related diseases. Respiratory symptoms have been related to the use of several domestic fuels, such as kerosene and other fuels in India. It is accountable for large number of COPD in the rural inhabitants in general and women in particular^42^–^45^. Main health effects from indoor pollution are respiratory symptoms, lung function reduction and decline, bronchial hyper-responsiveness and respiratory infections, some of which are also characteristics of COPD.

## D] DIET

There is usually a sequence in the emergence of chronic disease as the diet in developing countries becomes westernized. The “Westernized” diet of developed countries is characterized by excess of fat and free sugars and deficiency of complex carbohydrate foods-the main source of dietary fibers. A major factor that has greatly impacted the dietary pattern is urbanization in developing countries as a household can no longer depend on home grown produce any more. While rural communities depend on staple crops of cereals, tubers, vegetables and fruits, urbanization leads to increased consumption animal fat and sugar. This new diet pattern is perceived as a status symbol and readily accepted by other affluent communities. Within the urban setting, the food industry exerts substantial influence by promoting consumption of soft drinks, meat products, confectionary, snack foods, and other convenience foods rich in free sugars and fats. Bruney et al in a study showed that changes in dietary habits, such as increasing salt intake, decreasing intake of fruits and vegetables, and changing fatty acid consumption of the diet, were suggested to contribute to the rise in COPD mortality and morbidity.

The fact that not all patients with COPD are smokers but only 20 % of the smokers develop COPD^46^ has led to the alternative theories as to why some people appear to be more vulnerable to effects of cigarette smoke. Various studies have described the presence of nutritional abnormalities in patients with COPD. The most obvious clinical expression of these nutritional abnormalities is unexplained weight loss. Malnutrition contributes to respiratory muscle weakness resulting in increased frequency of hospitalization, Cor pulmonale and increased mortality. Nutrition depletion, as indicated by weight loss and loss of lean body mass, a common complication of advanced COPD (particularly, but not limited to, the emphysematous type). Low body weight or recent weight loss and in particular depleted lean body mass in patients with COPD have been shown to be independent predictors of mortality, poor outcomes after acute excaberations, hospital admission rates, and need for mechanical ventilation. The factors thought to contribute to nutrition depletion in these patients include elevated resting and activity-related energy expenditure, reduced dietary intake relative to energy expenditure, accelerated negative nitrogen balance, particularly during acute exacerbations of COPD, medication effects, and perhaps most importantly an elevated systemic inflammatory response.

## E] ALCOHOL

Global alcohol consumption has increased in recent decades, with most or all of this increase occurring in developing countries. Both average volume of alcohol consumption and patterns of drinking vary dramatically between sub regions. Average volume of drinking is highest in Europe and North America, and lowest in the Eastern Mediterranean. Patterns are most detrimental in Europe. Patterns are least detrimental in Western Europe (EURA) and the more economically established parts of the Western Pacific region (World Health Report 2002) Reports on the association between alcohol consumption and the prevalence of COPD vary. Heavy alcohol consumption was associated with respiratory symptoms and reduced lung function in a study by Lebowitz^47^ even when controlled for smoking. Smoking, however, was judged to be a far more important risk factor. The effect of passive smoking would have to be also considered in any heavy drinker, smoker or not, who consumed his or her favorite beverage in heavily tobacco-polluted bars or restaurants.

## F] SOCIOECONOMIC STATUS

Socioeconomic status may be assessed by using a series of indicators, which include income, education, type of work, house conditions, crowding index (family size with respect to house size). A low socioeconomic level is a risk factor for the development of emphysema and chronic bronchitis^48^. Strachan^49^ analyzing the 1979-1983 mortality rates in Great Britain, showed that, for both bronchitis-emphysema-asthma and trachea-bronchuslung cancer, the standardized mortality ratio increased with a decreasing socioeconomic status. An association between the prevalence of chronic bronchitis and a low socioeconomic status, even after adjusting for smoking and other risk factors was detected in Brazil in 1994. In Norway, Bakke et al.^50^ found adjusted odds ratios of spirometric airflow limitation of 5.2 and 1.8 for subjects who had completed only primary and secondary education, respectively, compared to university graduates. The socioeconomic status in early life might be a relevant risk factor for COPD as shown by a longitudinal analysis of the British Medical Research Council's national survey of health and development of the 1946 birth cohort^51^. At the age of 36 years, in both sexes, the presence of respiratory symptoms and the level of peak expiratory flow were independently associated with current indices of poor social circumstances and with poor environment at the age of 2 years.

## CONCLUSION

COPD and related conditions result in a huge burden in terms of health resource utilization as well as in overall costs worldwide. The striking increase in COPD prevalence globally cannot be primarily due to genetic factors, since it is occurring too rapidly, and therefore it must be occurring due to changes in environmental exposures. It seems that as a result of “Westernization package” changes in the lifestyle and environmental condition, we are seeing an increased susceptibility to the development of COPD. There are number of elements in this “package” including increased consumption of tobacco smoking amongst females and children, changing dietary pattern and increasing burden of industries and population leading to increased air pollution. All of these are associated with the increased risk of the disease, but none of which alone cannot explain the increase in prevalence. Thus it is important that we consider the “forest” of these changes that occur with westernization while doing studies on specific “trees”.
